# Supplementary material for: Disentangling locus of perceptual learning in the visual hierarchy of motion processing
Source: Sci Rep. 2019 Feb 7;9:1557. doi: 10.1038/s41598-018-37892-x (PMC6367332; doi:10.1038/s41598-018-37892-x)
Supplement: Supplementary file 1 — Supplementary Info [file 41598_2018_37892_MOESM1_ESM.docx]

**SUPPLEMENTARY INFORMATION**

**Title:** Disentangling locus of perceptual learning in the visual hierarchy of motion processing

Authors: Ruyuan Zhang and Duje Tadin

**Table of Contents**

Supplementary Figure 1: Learning curves of individual subjects

Supplementary Figure 2: Thresholds and percent of improvement of individual subjects

Figure S1. Individual learning curves for all subjects in the two training groups. Each line represents a learning curve of one subject. Other figure conventions are the same as in the Figure 4 in the main text.


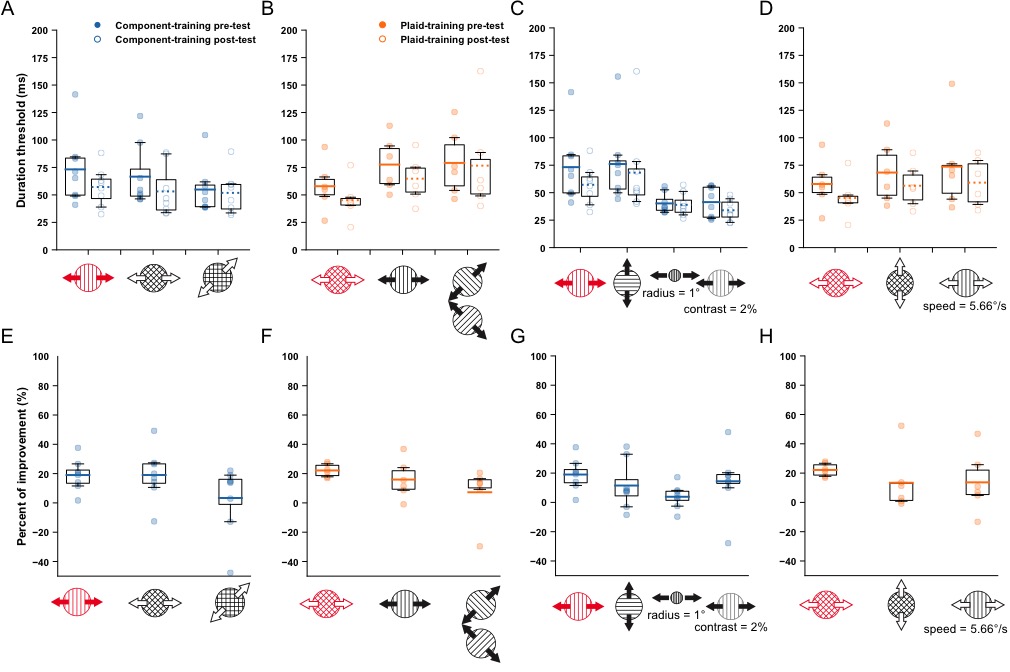


Figure S2. Duration thresholds and percent of improvement for all individual subjects, represented as scatter and box plots. Panels A-D depict duration thresholds at pre-test and post-test. Panels E-H depict percent of improvement for all training conditions (panels E-H correspond to panels A-D, respectively). Panels A, B, E and F correspond to the subplots E and F of Figure 5 in the main text. Panels C, D, G and H correspond to the subplots E and F of Figure 6 in the main text. In each panel, the shaded points represent the data for individual subjects. The lower and the upper boundaries of a box represent the 1^st^ and the 3^rd^ quartiles of the group data, respectively. The reach of whiskers denotes the 2.5^th^ and the 97.5^th^ percentiles of the group data. The horizontal lines in the box indicate the group averages, which correspond to the bar heights in the subplots E and F of Figures 5 and 6 in the main text. For panels A-D, the solid and the dashed lines represent averages for pre-test and post-test, respectively. Other figure conventions are the same as in Figures 5 and 6 in the main text.
