# Supplementary figures and images for: Disentangling locus of perceptual learning in the visual hierarchy of motion processing
Source: Sci Rep. 2019 Feb 7;9:1557. doi: 10.1038/s41598-018-37892-x (PMC6367332; doi:10.1038/s41598-018-37892-x)

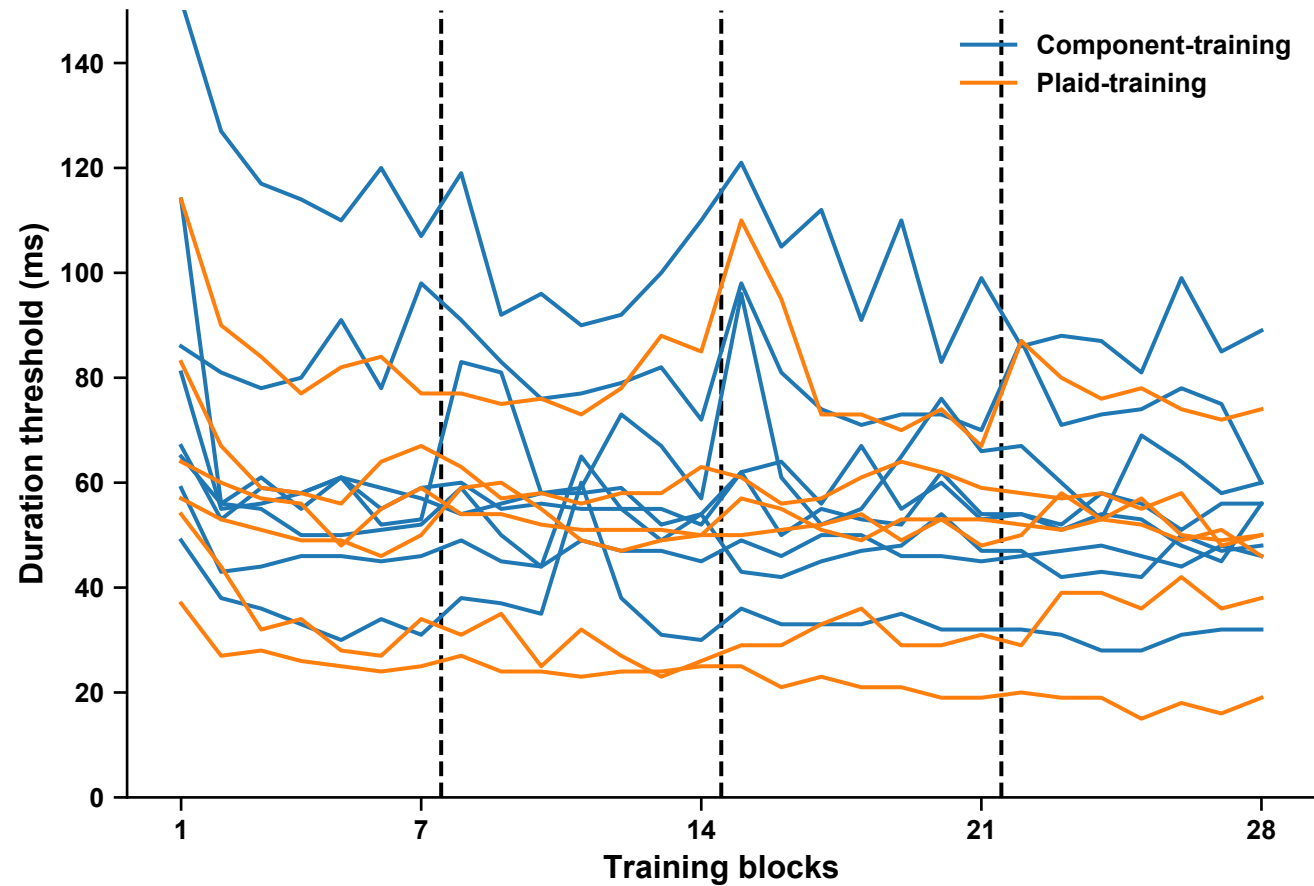

Supplement: Supplementary file 2 — Figure S1 [file 41598_2018_37892_MOESM2_ESM.pdf]

A

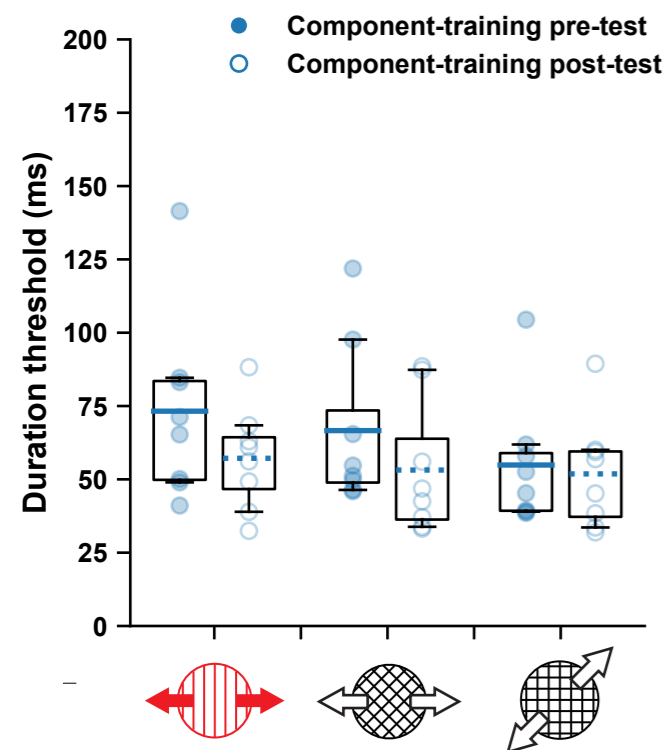

B

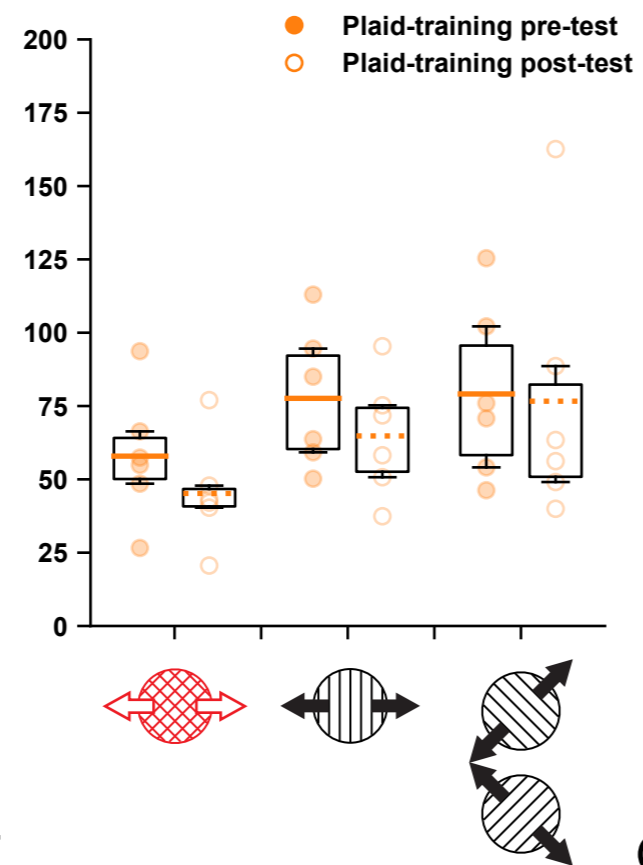

C

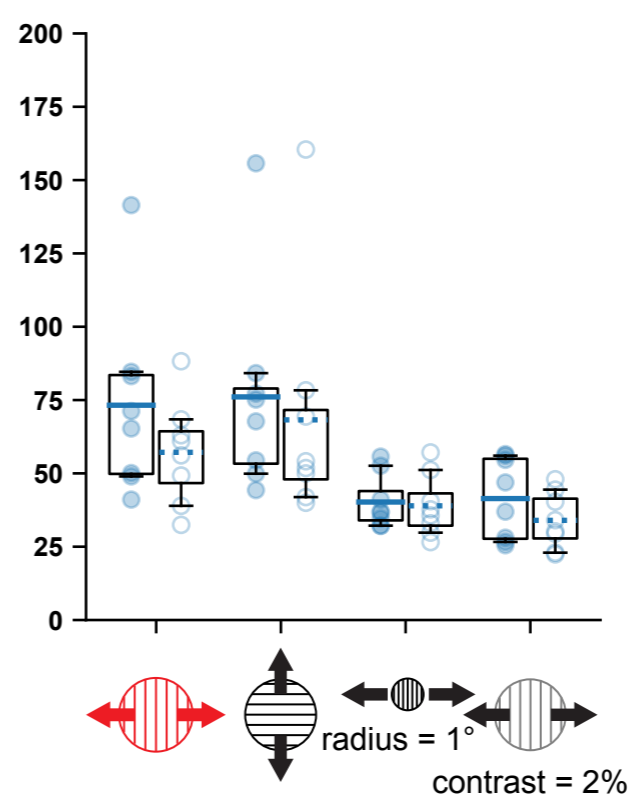

D

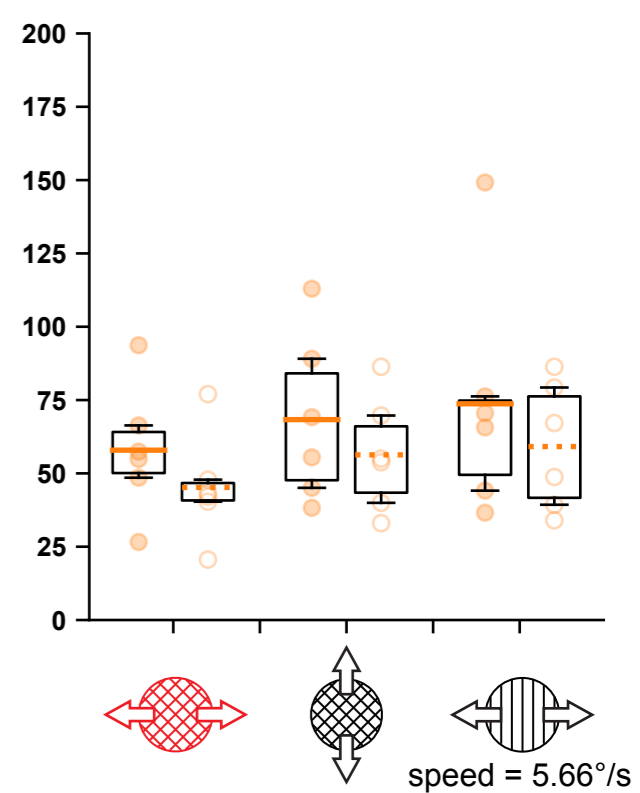

E

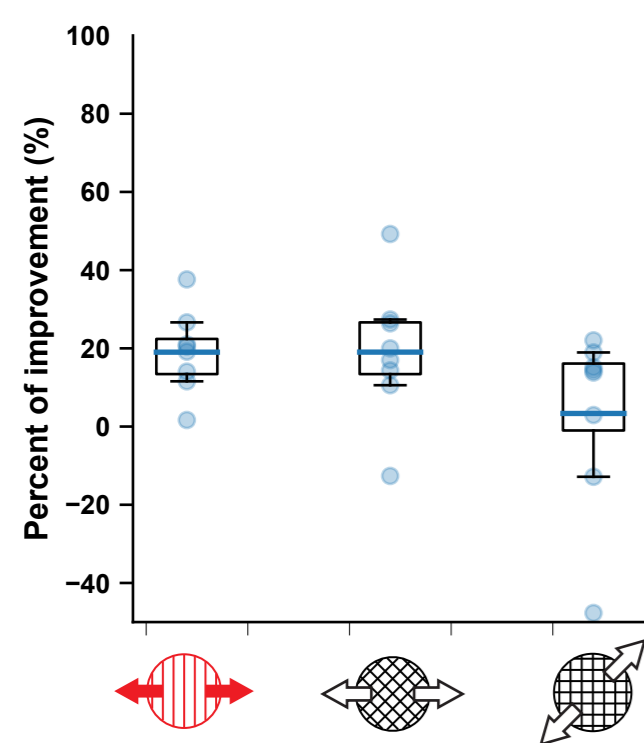

F

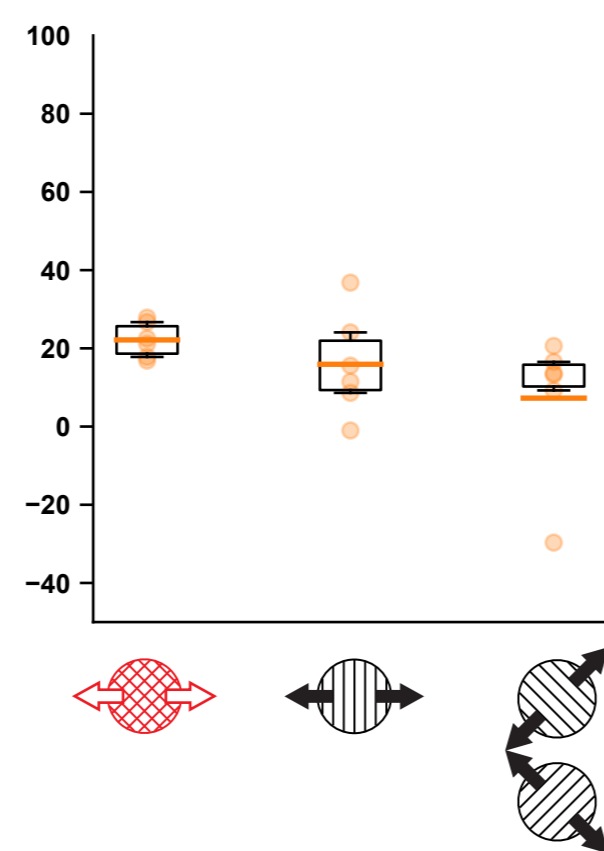

G

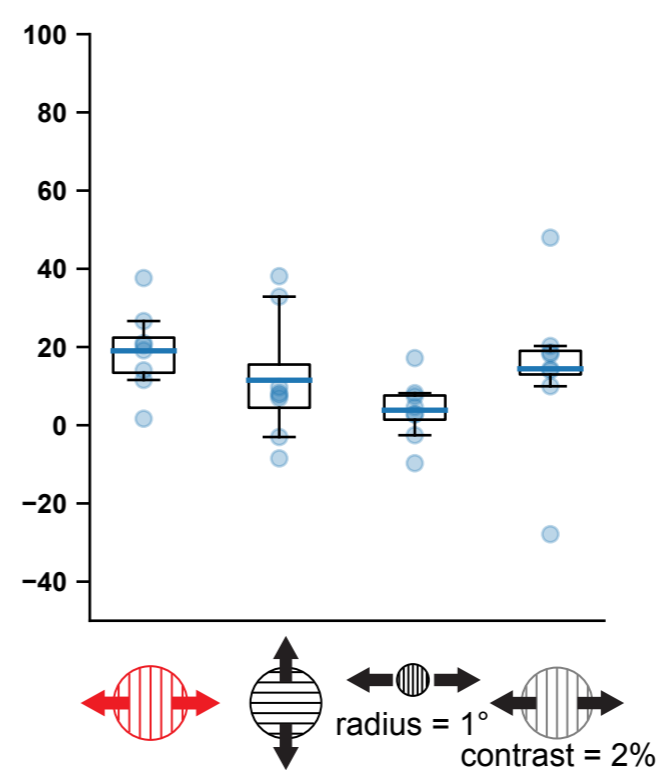

H

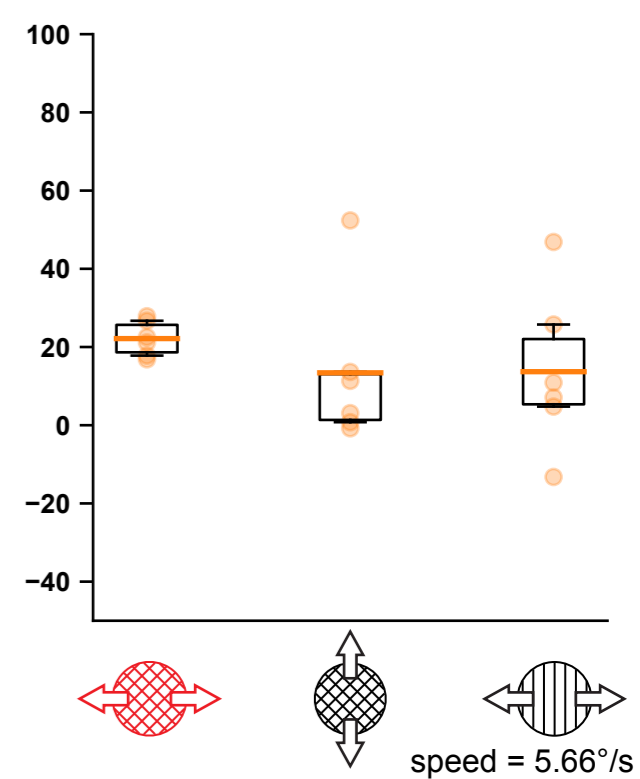

Supplement: Supplementary file 3 — Figure S2 [file 41598_2018_37892_MOESM3_ESM.pdf]
